# Health-related quality of life in women with breast cancer: a review of measures

**DOI:** 10.1186/s12885-021-09157-w

**Published:** 2022-01-15

**Authors:** Maribel Salas, Margaret Mordin, Colleen Castro, Zahidul Islam, Nora Tu, Michelle D. Hackshaw

**Affiliations:** 1 Epidemiology, Clinical Safety and Pharmacovigilance, Daiichi Sankyo, Inc., 211 Mount Airy Road, 1A-453, Basking Ridge, NJ 07920 USA; 2 CCEB/CPeRT, University of Pennsylvania Perelman School of Medicine, Philadelphia, PA USA; 3 RTI Health Solutions, Ann Arbor, MI USA; 4 RTI Health Solutions, Research Triangle Park, NC USA

**Keywords:** PROMs, Breast cancer

## Abstract

**Background:**

To identify and describe the breast cancer–specific health-related quality of life (HRQoL) instruments with evidence of validation in the breast cancer population for potential use in patients treated for breast cancer (excluding surgery).

**Methods:**

We conducted a systematic literature review using PubMed, Embase, and PsycINFO databases to identify articles that contain psychometric properties of HRQoL instruments used in patients with breast cancer. Relevant literature from January 1, 2009, to August 19, 2019, was searched. Articles published in English that reported psychometric properties (reliability, validity) of HRQoL instruments were identified.

**Results:**

The database search yielded 613 unique records; 131 full-text articles were reviewed; 80 articles presented psychometric data for instruments used in breast cancer (including generic measures). This article reviews the 33 full articles describing psychometric properties of breast cancer-specific HRQoL instruments: EORTC QLQ-C30, EORTC QLQ-BR23, FACT-B, FBSI, NFBSI-16, YW-BCI36, BCSS, QuEST-Br, QLICP-BR, INA-BCHRQoL, and two newly developed unnamed measures, one by Deshpande and colleagues (for use in India) and one by Vanlemmens and colleagues (for use among young women and their partners). The articles that described the EORTC QLQ-C30, QLQ-BR23, and FACT-B centered on validating translations, providing additional support for content validity, and demonstrating acceptability of electronic patient-reported outcome administration. Psychometric properties of the measures were acceptable. Several new measures have been developed in Asia with an emphasis on development on cultural relevance/sensitivity. Others focused on specific populations (i.e., young women with breast cancer).

**Conclusions:**

Historically, there have been limited options for validated measures to assess HRQoL of patients with breast cancer. A number of new measures have been developed and validated, offering promising options for assessing HRQoL in this patient population. This review supports the reliability and validity of the EORTC QLQ-C30 and FACT-B; new translations and electronic versions of these measures further support their use for this population.

## Background

Patient-reported outcomes (PROs) are defined as a “measurement of any aspect of a patient’s health status that comes directly from the patient (i.e., without the interpretation of the patient’s responses by a physician or anyone else)” [1]. Patient-reported outcome measures (PROMs) provide an opportunity for patients to indicate the impact of a disease and its treatment on their lives. Health-related quality of life (HRQoL) represents a patient’s physical, psychological, and social response to disease and therapy and is one type of PRO [2]. PROs can provide additional information to help with treatment approval, reimbursement, and selection/dosing decisions; management of medication side effects; health monitoring; and patient-provider decision-making.

Breast cancer is the most commonly occurring cancer in women, with an estimated 2 million new cancer cases diagnosed globally in 2018 [3]. Advanced breast cancer has been described as a generally incurable, yet treatable disease, and the primary goals of treatment are to reduce symptom burden, maintain quality of life (QoL), and prolong survival [4, 5]. Treatment for patients with advanced disease includes neoadjuvant chemotherapy, surgery, postsurgical radiation therapy, and systemic adjuvant therapy (including hormone therapy for those with hormone receptor-positive breast cancers). Understanding the impact of treatment on patients’ HRQoL outside clinical trials can provide useful information for patients and clinicians in making treatment decisions.

Several PROMs have been used to assess patients with breast cancer. PROMs are questionnaires that capture patients’ feelings and functioning in a structured manner and consist of items and corresponding response options; When developed and validated according to international guidelines, PROMs can provide reliable and valid patient assessment.

A systematic literature review by Nguyen et al. [6] indicated that the European Organization for Research and Treatment of Cancer (EORTC) Breast Cancer–Specific Quality of Life Questionnaire-23 item (QLQ-BR23) and the Functional Assessment of Cancer Therapy-Breast (FACT-B) are the only HRQoL questionnaires that have been developed specifically for patients with breast cancer facing different disease stages and treatments. Both tools act as supplements to their general cancer questionnaires, the EORTC Quality of Life Questionnaire, Version 3.0 (QLQ-C30) and the FACT-G, respectively. Given recent developments in breast cancer treatment, we sought to determine whether additional valid and reliable HRQoL measures are available in the public domain. Specifically, we aimed to identify breast cancer–specific HRQoL measures with evidence of validation in the breast cancer population for potential use in patients underwent systemic treatment for breast cancer (excluding surgery and radiotherapy).

As HRQoL measures are focused on patients’ overall health and well-being, regional characteristics and traditions may be included. This review focused on identifying HRQoL measures regardless of whether or not they were region specific.

## Methods

### Literature search

The literature review was conducted on August 19, 2019, in the PubMed, Embase, and PsycINFO databases. Table 1 presents the search strategy used for PubMed; the key words used were translated for each of the individual databases. The search focused on the past 10 years (January 1, 2009–August 19, 2019), was limited to publications written in English, and excluded commentaries, letters to editors, editorials, book chapters and case reports because they did not contain detailed information on the psychometric properties of the instruments.

**Table 1:** PubMed Search Strategy

Search No.	Search Terms	No. of Articles
**Disease**		
#1	“Breast Neoplasms”[Majr] OR breast neoplasm*[Title] OR breast cancer*[Title] OR breast carcinoma*[Title] OR breast tumor*[Title] OR breast tumour*[Title] OR mammary cancer*[Title] OR mammary carcinoma*[Title] OR mammary neoplasm*[Title] OR mammary tumor*[Title] OR mammary tumour*[Title] OR “cancer of the breast”[Title] OR breast malignan*[Title] OR mammary malignan*[Title]	**123,621**
**Quality of life**		
#2	#1 AND ((“Quality of Life”[Majr] OR “quality of life”[Title] OR “life quality”[Title/Abstract] OR QoL[Title/Abstract] OR hrql[Title/Abstract] OR hrqol[Title/Abstract] OR “EORTC-QLQ-C30”[Title/Abstract] OR “FACT-B”[Title/Abstract]) NOT (“Quality-Adjusted Life Years”[Mesh] OR “quality adjusted life year”[Title] OR “quality adjusted life years”[Title] OR QALY[Title] OR health utilit*[Title] OR HUI[Title] OR “standard gamble”[Title] OR “time trade off”[Title] OR “time tradeoff”[Title] OR TTO[Title]))	**2260**
**Validity**		
#3	#2 AND (“Psychometrics”[Mesh] OR valid*[Text Word] OR reliable*[Text Word] OR reliability[Text Word] OR psychometric*[Text Word] OR (concurrent[Text Word] AND validity[Text Word]) OR (divergent[Text Word] AND validity[Text Word]) OR responsiveness[Text Word] OR responder*[Text Word] OR correlation coefficient*[Text Word] OR correlation co-efficient*[Text Word])	**311**
**Exclusions**		
#4	“Animals”[Mesh] NOT “Humans”[Mesh]	**1,144,986**
#5	“Comment”[Publication Type] OR “Letter”[Publication Type] OR “Editorial”[Publication Type]	**753,935**
**Totals**		
#6	(#3 NOT (#4 OR #5))	**310**

Limits: 2009-present; English; Humans; No comments, letters, or editorials

### Literature review

Unique records that were identified across the three databases were reviewed in accordance with prespecified inclusion criteria. Studies were required to include patients (aged ≥18 years) with breast cancer who were treated with a pharmaceutical intervention and to assess a psychometric property of an HRQoL-focused PROM. Psychometric properties of interest included reliability (internal consistency, Cronbach alpha, test-retest), validity (content, convergent, divergent), and responsiveness. Psychometric properties for different modes of administration (e.g., electronic PRO [ePRO]) or for translations were included. Reasons for exclusion were populations receiving surgery or radiation, studies focused on HRQoL of treatment efficacy only, and studies only considering caregiver burden. References of relevant review articles were reviewed for any pertinent articles not identified in the original search. Two investigators reviewed the abstracts and selected abstracts that fulfilled the inclusion criteria. Any disagreement among investigators was discussed and final decision was done based on consensus.

During level 1 screening (titles and abstracts), studies that did not meet criteria were excluded. Full texts of included studies were reviewed (level 2 screening) using the same relevance criteria applied at level 1. Upon completion of level 2, an additional criterion was added to focus the review on breast cancer–specific HRQoL instruments only.

Information regarding reliability (internal consistency, Cronbach alpha, test-retest) and validity (content, convergent, divergent) were extracted from the studies. These psychometric properties were analyzed in accordance with prespecified thresholds of significance (e.g., Cronbach alpha > 0.7). In addition, item content of the instruments was reviewed.

## Results

**Fig. 1:**
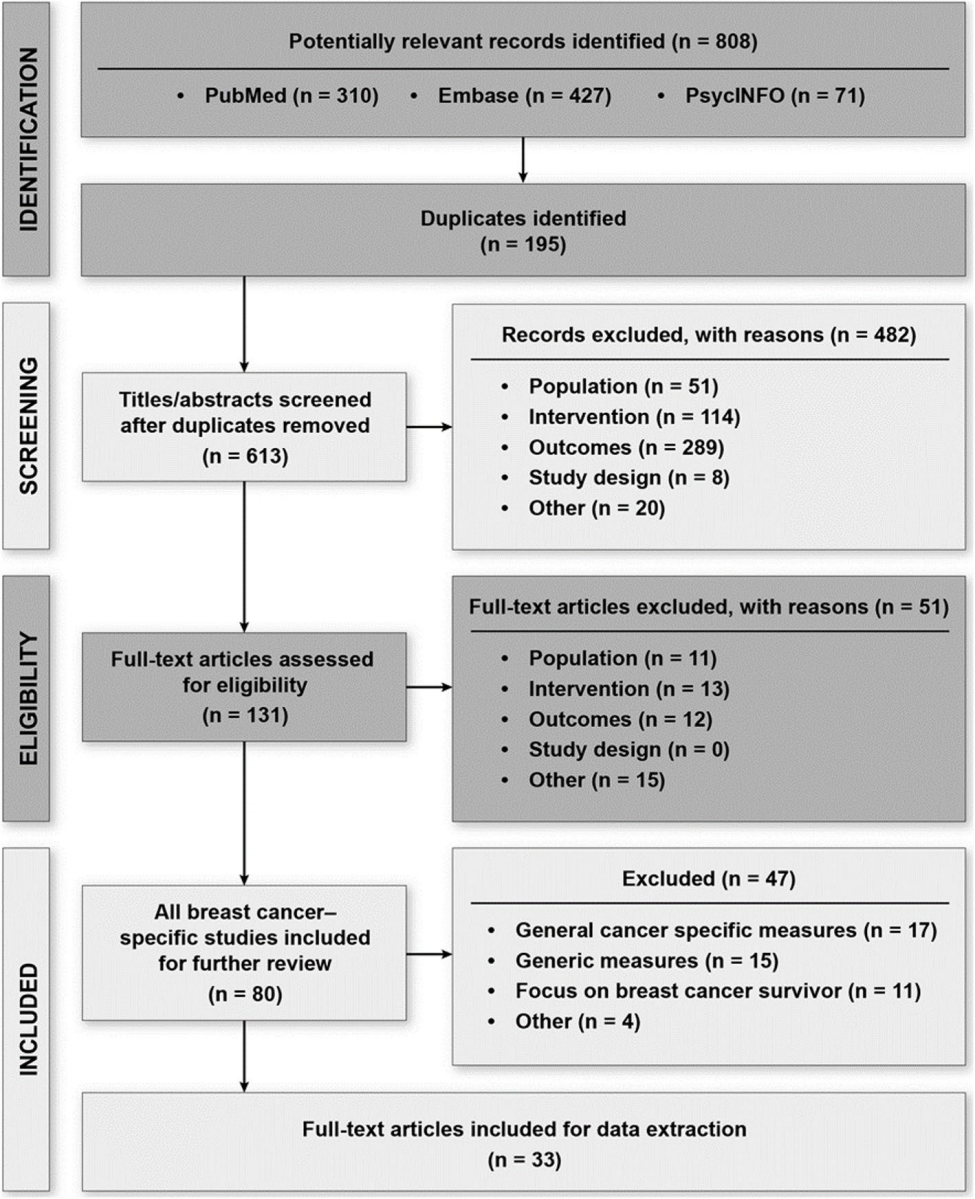
PRISMA Diagram

Figure 1 summarizes the literature review, which identified 613 unique records for level 1 screening, of which 131 full-text articles were reviewed; 80 articles presented psychometric properties for identified PROMs used in breast cancer. This review focuses on the 33 that described psychometric properties of breast cancer–specific HRQoL instruments.

Psychometric properties of 12 PROMs were identified: EORTC QLQ-C30, EORTC QLQ-BR23, FACT-B, Functional Assessment of Cancer Therapy-Breast Symptom Index (FBSI), National Comprehensive Cancer Network-Functional Assessment of Cancer Therapy-Breast Cancer Symptom Index-16 (NFBSI-16), Young Women with Breast Cancer Inventory (YW-BCI36), Breast Cancer Symptom Scale (BCSS), QuEST Breast Cancer Questionnaire (QuEST-Br), Quality of Life Instruments for Cancer Patients-Breast Cancer (QLICP-BR), Indonesian Breast Cancer Health-Related Quality of Life (INA-BCHRQoL), and two new unnamed measures [7–9]. For each identified PROM, Table 2 provides an overview of the measure’s purpose, the domains assessed and the number of items. Table 3 provides an overview of concepts addressed for each PROM. Table 4 provides an overview of the psychometric articles (instrument, objective, population), and Table 5 provides psychometric qualities of the identified instruments.

**Table 2:** Overview of Identified Measures

Instrument	Purpose	Domains	No. of Items
European Organization for Research and Treatment of Cancer (EORTC) Quality of Life Questionnaire, Version 3.0	Cancer-specific questionnaire designed to measure QoL in the cancer population	9 domains: physical, role, cognitive, emotional, social, fatigue, pain, nausea and vomiting, global health status and quality of life	30
EORTC Breast Cancer–Specific Quality of Life Questionnaire	Designed to measure QoL in the breast cancer population at various stages and with patients with differing modalities	5 domains: therapy side effects, arm symptoms, breast symptoms, body image, sexual functioning	23
Functional Assessment of Cancer Therapy-Breast (FACT-B)	Specific to breast cancer patients	6 domains: physical well-being (PWB), social/family well-being, emotional well-being (EWB), functional well-being (FWB), relationship with doctor, additional concerns	37
Functional Assessment of Cancer Therapy-Breast Symptom Index (FBSI)	Symptom specific instrument for breast cancer patients	8 items extracted from the FACT-B as follows: PWB (4 items), EWB (1 item), and FWB (1 item) subscales, as well as the breast cancer subscale (2 items) of the FACT-B	10
National Comprehensive Cancer Network-Functional Assessment of Cancer Therapy-Breast Cancer Symptom Index-16	Builds upon the original FBSI	3 domains: Disease-Related Symptom—9 items; Treatment Side Effect—4 items; and General Function and Well-Being—3 items	16
Breast Cancer Symptom Scale	Designed to assess breast cancer symptoms	NR	NR
QuEST Breast Cancer Questionnaire	Designed for use in routine clinical practice, aiming to provide assessment tool for patients with breast cancer receiving chemotherapy treatments	5 physical and 3psychosocial function scales plus disease-specific individual symptom items	NR
Quality of life Instruments for Cancer Patients-Breast Cancer	Chinese-specific QoL instrument combining general module with breast cancer–specific module	5 domains: physical, psychological, social, social support and safety, specific (breast symptoms, upper body effect, physical, and psychologic effect)	39 (32 general; 7 breast cancer)
Indonesian Breast Cancer Health-Related Quality of Life	Developed, instead of translated using forward and backward translation to provide a questionnaire that is suitable for Indonesian patients with breast cancer	4 domains: physical; cognitive and psychological, social, spiritual	41
Young Women with Breast Cancer Inventory	Measures the impact of breast cancer on the quality of life of young women (aged < 45 years) with nonmetastatic disease	8 domains: (1) feeling of couple cohesion, (2) negative affectivity and apprehension about the future, (3) management of child(ren) and of everyday life, (4) sharing with close relatives, (5) body image and sexuality, (6) financial difficulties, (7) deterioration of relationships with close relatives, and (8) career management	36
Not named (Indian Breast Cancer QoL measure; Deshpande et al. [7])	Designed to assess QoL among patients in India with breast cancer	11 domains	24
Not named (Nonmetastatic Young Women and their partners measure; Vanlemmens et al. [9] and [8])	Designed to measure the impact of breast cancer on the QoL of young women (aged < 45 years) with nonmetastatic disease	8 relevant areas: psychological, physical, family, social, couple and sexuality, domestic, professional, and economic	36

*NR* not reported, *QoL* quality of life

**Table 3:** Content Mapping Across Patient-Reported Outcome Measures

	QLQ-C30	QLQ-BR23	FACT B	FBSI	NFBSI-16	BCSS	QuEST Br	QLICP-BR	INA-BCHRQoL	YW-BCI36	Desphande [7]	Vanlemmens et al. [8, 9]
Physical	✓		✓	✓	✓		✓	✓	✓			✓
Role	✓											
Cognitive	✓								✓			
Emotional	✓		✓	✓	✓		✓					
Social	✓		✓				✓	✓	✓			✓
Fatigue	✓											
Pain	✓											
Nausea and vomiting	✓											
Global HS	✓											
QoL	✓											
Therapy side effects		✓			✓							
Arm symptom		✓										
Breast symptoms		✓		✓	✓	✓	✓	✓				
Body image		✓								✓		
Sexual function		✓										
Functional well-being			✓	✓	✓							
Relationships with doctor/others			✓							✓		
Additional concerns			✓									
Function/well-being					✓							
Social support								✓				
Psychological								✓	✓			✓
Spiritual									✓			
Couple cohesion										✓		✓
Negative affect/worry future										✓		
Manage kids/everyday life										✓		✓
Sharing with relatives										✓		
Sexuality										✓		
Financial										✓		✓
Career										✓		✓

*BCSS* Breast Cancer Symptom Scale, *FACT-B* Functional Assessment of Cancer Therapy-Breast, *FBSI* Functional Assessment of Cancer Therapy-Breast Symptom Index, *INA-BCHRQoL* Indonesian Breast Cancer Health-Related Quality of Life, *NFBSI-16* Network-Functional Assessment of Cancer Therapy-Breast Cancer Symptom Index-16, *QLICP-BR* Quality of Life Instruments for Cancer Patients-Breast Cancer, *QLQ-BR23* Breast Cancer–Specific Quality of Life Questionnaire, *QLQ-C30* Quality of Life Questionnaire, Version 3.0, *QoL* quality of life, *QuEST Br* QuEST Breast Cancer Questionnaire, *YW-BCI36* Young Women with Breast Cancer Inventory

**Table 4:** Overview of Psychometric Articles Identified

Reference	Primary Instrument	Objective	Population	Sample Size and Age
**EORTC**				
Alawadhi et al. [10]	EORTC QLQ-BR23/EORTC QLQ-C30	▪ Assess the psychometric characteristics of both questionnaires using the responses of a larger sample of Arab women	▪ Women attending follow-up clinic appointment for chemotherapy	▪ 348 women ▪ Mean (SD) age: 48.3 (10.3) y
Bener et al. [11]	EORTC QLQ-C30	▪ Assess psychometric properties of the Arabic version for patients in Qatar with breast cancer	▪ Patients with breast cancer identified from hospital-based disease registry (ICD-10 code C50)	▪ 678 patients with breast cancer ▪ Mean (SD) age: 47.7 (10.2) y
Bjelic-Radisic et al. [12]	EORTC QLQ-BR23/QLQ-BR45	▪ Phase I-III study updating the EORTC QLQ-BR23	▪ Patient data that were included in an extensive literature systematic review to identify relevant QoL issues; interviews with patients and health care providers	▪ 250 patients from 12 countries
Cerezo et al. [13]	EORTC QLQ-BR23	▪ Validate the Mexican-Spanish version EORTC QLQ-BR23	▪ Women with biopsy-proven breast carcinoma	▪ 234 women were included ▪ Mean (SD) age: 52.9 (11.8) y
El Fakir et al. [14]	EORTC QLQ-BR23	▪ Test the reliability and validity of the Moroccan Arabic version	▪ Subjects were eligible if they were aged at least 18 y, had a confirmed diagnosis of breast cancer, and spoke Moroccan Arabic	▪ 105 patients participated (37 for test-retest) ▪ Mean age: 48 y
Keilmann et al. [15]	EORTC QLQ-C30	▪ Investigate the reliability of a German, tablet-based version of the EORTC QLQ-C30	▪ Female patients with breast cancer in adjuvant and metastatic situation	▪ 106 patients
Michels et al. [16]	EORTC QLQ-BR23/EORTC QLQ-C30	▪ To validate and assess reliability and understanding of the EORTC QLQ-C30 and the EORTC QLQ-BR23 (in Brazil)	▪ Women aged between 27 and 90 y, diagnosed with breast cancer, treated or in treatment, at any disease stage	▪ 100 women ▪ Mean (SD) age: 56.5 (12.4) y
Shuleta- Qehaja et al. [17]	EORTC QLQ-C30	▪ Evaluate the validity and reliability of the QLQ-C30 in Albanian women with breast cancer	▪ Sample of patients with breast cancer	▪ 62 women ▪ Mean (SD) age: 50 (10.9) y
Simons [18]	EORTC QLQ-C30	▪ Assess content validity for signs and symptoms component	▪ Women with locally advanced or metastatic breast cancer were required to have received prior treatment with an anthracycline, a taxane, and capecitabine	▪ 283 women provided 1068 questionnaires ▪ Age- NR
Snyder et al. [19]	EORTC QLQ-C30	▪ Test the replicability of the QLQ-C30 cutoff scores from previous study	▪ Inclusion criteria: diagnosis of breast cancer, age at least 20 y, awareness of cancer diagnosis, and Eastern Cooperative Oncology Group (ECOG) performance status of 0–3	▪ 408 Japanese ambulatory patients with breast cancer ▪ Mean age: 56 y
Tan et al. [20]	EORTC QLQ-BR23/EORTC QLQ-C30	▪ Validate EORTC QLQ-C30 and EORTC QLQ-BR23 questionnaires ▪ Measure HRQoL of women with breast cancer in Singapore during their first 4 y of postdiagnosis and treatments	▪ Women older than 21 y with breast cancer stage 0 to 3A and in the first 4 y of postinterventions were recruited	▪ 170 patients participated in the study ▪ Mean ± SD age: 54 ± 9 y
Wallwiener et al. [21]	EORTC QLQ-C30 and FACT-B	▪ Analyze acceptance of an electronic patient-reported outcome survey tool in patients with breast cancer	▪ Patients with breast cancer in adjuvant and metastatic situation	▪ 110 patients ▪ Mean age: 52.4 y
Wallwiener et al. [22]	EORTC QLQ-C30	▪ Analyze the reliability of a tablet-based measuring application for EORTC QLQ-C30 in German	▪ Female patients with adjuvant and metastatic breast cancer	▪ 106 women ▪ Arm A (*n* = 53) completed tablet, then paper version ▪ Arm B (*n* = 53) completed paper, then tablet version ▪ Age: NR
Zhang et al. [23]	EORTC QLQ-BR23	▪ Evaluate the reliability and validity of the Chinese version	▪ Patients were included if they were aged > 18 y and had histological conformation of breast cancer	▪ 294 outpatients with breast cancer ▪ Mean age: 53.1 y
**FACT-B**				
Algamdi and Hanneman [24]	FACT-B	▪ Test reliability of the Arabic version of the FACT-B in women with breast cancer	▪ Community-dwelling adults (aged 18–75 y) Arabic-speaking persons diagnosed with cancer (patients with breast cancer completed the FACT-B)	▪ 29 women with breast cancer ▪ Age - NR
Cheung et al. [25]	FACT-B	▪ Compare the measurement precision and related properties between EQ-5D-5L and FACT-B questionnaires in assessing breast cancer patients	▪ Asian patients with breast cancer	▪ Observational study of 269 Singaporean patients with breast cancer ▪ Age: NR
Jarkovsky et al. [26]	FACT-B	▪ Validate FACT-B translation to the Czech language	▪ Consecutive patients with breast cancer	▪ 106 patients with breast cancer; 83 selected for psychometric testing
Kobeissi et al. [27]	FACT-B	▪ Translate, adapt, and face-validate the FACT-B into Arabic, specifically in the context of the Lebanese culture	▪ Lebanese females who met the following inclusion criteria: breast cancer diagnosis, patient follow-up by oncology clinics, patient awareness of their diagnosis, patient willingness to share their experiences	▪ Face-to-face interviews with individual patients with breast cancer (*n* = 33) and 2 focus groups (4 women/group) ▪ Mean (SD) age: 47.2 (11.8) y
Lee et al. [28]	FACT-B	▪ Compare the measurement properties between the EQ-5D-5L and the FACT-B	▪ Singaporean, histologically confirmed patients with breast cancer who were aged at least 21 y	▪ 269 women ▪ Mean (SD) age: 52.1 (9.9) y
Matties et al. [29]	FACT-B	▪ Analyze the reliability of tablet-based measurement of FACT-B in the German language	▪ Paper- and tablet-based questionnaires were completed by 106 female adjuvant and patients with metastatic breast cancer	▪ Arm A (*n* = 53) completed tablet, then paper version ▪ Arm B (*n* = 53) completed paper, then tablet version ▪ Age- NR
Ng et al. [30]	FACT-B	▪ Examine the measurement properties of and comparability between the English and Chinese versions of the FACT-B in Singaporean patients with breast cancer	▪ Histologically confirmed breast cancer, at least 21 y old, ability to understand Chinese or English or both	▪ 271 patients in the analysis ▪ Mean (SD) age: ­ English: 50.7 (9.7) y ­ Chinese: 54.6 (9.8) y
Patoo et al. [31]	FACT-B	▪ Validate the FACT-B in a sample of Iranian women with breast cancer	▪ Breast cancer diagnoses, outpatients or inpatients with a pathologic diagnosis of cancer with any type or stage	▪ 300 women ▪ Mean age: 47.27 y
**FBSI**				
Lee et al. [32]	FBSI	▪ Examine the measurement properties of and comparability between the English and the Chinese versions of the FBSI and to compare it with its parent instrument, the FACT-B, in Singaporean patients with breast cancer	▪ Histologically confirmed breast cancer, at least 21 years old, ability to understand Chinese or English or both	▪ 271 patients in the analysis ▪ Mean (SD) age: ­ English: 50.7 (9.7) y ­ Chinese: 54.6 (9.8) y
**NFBSI-16**				
Garcia et al. [33]	NFBSI-16	▪ Develop a new version of the FBSI that is in accordance with the FDA guidance for patient-reported outcome measures, provide assessment on a symptom level, and improve upon the original FBSI by emphasizing the patient input	▪ 18 y old and diagnosed with stage III or IV breast cancer. They must have had experience with chemotherapy for at least 2 cycles (or 1 month with noncyclical chemotherapy)	▪ 52 patients with stage III or IV breast cancer ▪ Mean age: 54 y
Krohe et al. [34]	NFBSI-16	▪ Evaluate content validity of the NFBSI-16 and the PROMIS Physical Function SF10b	▪ Adult man or woman (≥18 y of age); clinician confirmation of metastatic or locally advanced breast cancer not amenable to curative treatment by surgery or radiotherapy; clinician confirmation of hormone-receptor positive (HR+; estrogen receptor positive and/or progesterone receptor positive) and human epidermal growth factor receptor-2–negative (HER2-) breast cancer	▪ 15 cognitive debriefing interviews ▪ Mean (SD) age: 66.0 (12.4)
**YW-BCI36**				
Christophe et al. [35]	Young Women with Breast Cancer Inventory	▪ Validate, in a large sample of patients, a questionnaire specifically measuring the subjective experience of the disease and its treatment in young women (aged < 45 y when diagnosed) living with a nonmetastatic breast cancer and the repercussions of the disease and its treatment they perceive in their daily life	▪ Aged < 45 y at their diagnosis for a nonmetastatic breast cancer, had received or were receiving chemotherapy	▪ 546 patients ▪ Mean (SD) age: 40.64 (6.21) y
**Breast Cancer Symptom Scale**				
Horigan et al. [36]	Breast Cancer Symptom Scale	▪ Determine issues important to patients with breast cancer	▪ Web-based survey from patients included in the NexCura information resource	▪ *N* = 1072 patients ▪ Median age: 53 y
**QLICP-BR**				
Wan et al. [37]	Quality of Life Instruments for Cancer Patients-Breast Cancer	▪ Develop and validate a QoL instrument for patients with breast cancer in China	▪ Breast cancer inpatients at any stages and treatments	▪ Inpatients (*N* = 186) with breast cancer answered the questionnaires at the time of admission to the hospital. 166 were entered to participate in a second assessment the following day or the second day after hospitalization to evaluate test-retest reliability. 94 cases were sampled randomly and assessed a third time after treatment to evaluate responsiveness. ▪ Mean (SD) age: 48.5 (10.1) y
**QuEST-Br**				
Harley et al. [38]	QuEST-Br	▪ Adapt previously existing HRQoL measures to generate a tool for use in routine clinical practice	▪ Patients in this study were attending outpatient clinics for review or chemotherapy treatment for breast cancer	▪ *N* = 145 patients with breast cancer ▪ Mean (SD): 51 (11.6) y
**INA-BCHRQoL**				
Saptaningsih et al. [39]	Indonesian Breast Cancer Health-Related Quality of Life	▪ Develop a new questionnaire in order to capture not only patients’ physical, cognitive, and psychological aspects but also the spiritual aspect	▪ Women with a diagnosis of breast cancer stage I to IIIA, confirmed by pathology and anatomy assessment or cytology test, who received surgery and combined with fluorouracil, doxorubicin, and cyclophosphamide (FAC)– or taxan-based chemotherapy	▪ *N* = 24 patients ▪ Age: 83% ≥40 y
**Unnamed**				
Deshpande et al. [7]	Newly developed, unnamed measure	▪ Develop and validate a patient-reported questionnaire to assess the QoL outcomes of Indian breast cancer patients	▪ Patients with breast cancer irrespective staging of cancer and type of therapy	▪ *N* = 30 patients ▪ Age: NR
Vanlemmens et al. [9]	Newly developed, unnamed measure	▪ Analyze the quality of life of young women (< 45 y) with nonmetastatic breast cancer and their partners,	▪ Young patients (aged < 45 y at the time of diagnosis) with nonmetastatic breast cancer and living with a partner were targeted	▪ *N* = 69 couples ▪ Age: NR
Vanlemmens et al. [8]	Unnamed measure (same as in 2009 publication)	▪ Create a particular and specific inventory for measuring the impact of breast cancer on the quality of life of young women (aged < 45 y) with nonmetastatic disease and the quality of life of their partners; this work presents the psychometric validation	▪ Women aged < 45 y when diagnosed and treated or been treated by chemotherapy for a nonmetastatic breast cancer and partners	▪ *N* = 546 patients and *n* = 499 partners ▪ Age: NR

*EORTC* European Organization for Research and Treatment of Cancer, *EQ-5D-5L* 5-level EuroQoL Group’s 5-dimension, *FACT-B* Functional Assessment of Cancer Therapy-Breast, *FBSI* Functional Assessment of Cancer Therapy-Breast Symptom Index, *FDA* US Food and Drug Administration, *HRQoL* health-related QoL, *ICD-10* International Classification of Diseases, Tenth Edition, *NFBSI-16* Network-Functional Assessment of Cancer Therapy-Breast Cancer Symptom Index-16, *NR* not reported, *PROMIS* Patient-Reported Outcomes Measurement Information System, *QLQ-BR23* Breast Cancer–Specific Quality of Life Questionnaire-23 item, *QLQ-BR45* Breast Cancer–Specific Quality of Life Questionnaire-45 item, *QLQ-C30* Quality of Life Questionnaire, Version 3.0, *QoL* quality of life, *QuEST-Br* QuEST Breast Cancer Questionnaire, *SD* standard deviation

**Table 5:** Psychometric Qualities of Identified Instruments

Instrument Reference	Reliability	Validity
**EORTC QLQ-BR23**		
Alawadhi et al. [10]	▪ The intraclass correlation for the test-retest statistic and the internal consistency values for the multi-item scales was > 0.7 ▪ With the exception of the pain subscale, all items met the item internal consistency criterion of > 0.4 correlation with the corresponding scale.	▪ The QLQ-BR23 performed better than the QLQ-C30 for item discriminant validity ▪ The scale scores discriminated between patients at different disease stages and between sick and well populations.
Bener et al. [11]	▪ 6 of the 9 subscales met the standards of reliability, with coefficients ranging from 0.55 to 0.89	▪ Advanced breast cancer stages of III-IV had significantly higher symptomatic scores than those in early stages for the physical function, cognitive, fatigue, insomnia, appetite loss, constipation, and financial difficulties. ▪ Correlation coefficients between each item ranged from − 0.113 to 0.960, and item 21 (tense) and item 23 (irritable) had strongest negative correlations with their corresponding emotional functioning subscale, whereas items 29 (physical condition) and 30 (overall QoL) had the strongest positive correlation with Global Health/QoL subscale. ▪ Item 6 (limited work) showed a higher correlation with fatigue (r = 0.749). ▪ Item 19 (pain interfered with daily activities) of the pain subscale had higher correlations with physical functioning, role functioning, and fatigue subscales.
Bjelic-Radisic et al. [12]	▪ NR	▪ Phases 1 and 2 results indicated the need to supplement the original QLQ-BR23, with additional items related to newer therapeutic options. ▪ The phase 3 study recruited a total of 250 patients from 12 countries. After the qualitative and quantitative analyses, the final updated phase 3 module contained a total of 45 items: 23 items from the QLQ-BR23 and 22 new items. The new items contain two multi-item scales: target symptom scale (20 items) and satisfaction scale (2 items). The target symptom scale can be further divided into 3 subscales: endocrine therapy scale, endocrine sexual scale, and skin/mucosa scale.
Cerezo et al. [13]	▪ Cronbach alpha of all multi-item scales showed values ≥0.7, except for Cognitive and Breast symptoms scales (0.52 and 0.65, respectively)	▪ Convergent and divergent validity was adequate ▪ Patients with early stages (*n* = 77) showed better functional scores and lower symptoms scores than patients with locally advanced breast cancer (*n* = 157) ▪ Score means variation after responsiveness analysis demonstrated high sensitivity to change after breast cancer surgery
El Fakir et al. [14]	▪ Cronbach alpha coefficient were all > 0.7, except for breast symptoms and arm symptoms	▪ All items exceeded the 0.4 criterion for convergent validity, except items 20 and 23 related to pain and skin problems in the affected breast, respectively
Keilmann et al. [15]	▪ Statistic differences could not be seen in the majority of the single items (27/30) nor in one of the scales, investigating the parallel form reliability; the test of consistency showed in 29 of 30 single items and 12 of 15 scales statistically significant correlations	▪ NR
Michels et al. [16]	▪ Cronbach alpha for the EORTC QLQ-C30 ranged from 0.72 to 0.86 and from 0.78 to 0.83 for the EORTC QLQ-BR23	▪ Most questions were confirmed in the confirmatory factorial analysis ▪ In the construct validity analysis, the questionnaires were capable of differentiating patients with or without lymphedema, apart from the symptom scales of both questionnaires ▪ Both questionnaires presented a significant correlation in most domains of the SF-36 in the convergent validity analysis ▪ Only a few criticisms were reported concerning questions, and the mean grade of understanding was high (QLQ-C30 = 4.91 and QLQ-BR23 = 4.89)
Shuleta-Qehaja et al. [17]	▪ Cronbach alpha ranged from 0.54 for the cognitive functioning scale to 0.96 for the global health quality of life (GH/QoL) scale	▪ In multitrait scaling analysis, the strength of Spearman correlations between an item and its own subscale was ≥0.40, with the exception of item 5 (*Ρ* = 0.22); results for item discriminant validity were satisfactory, with the exception of item 5, which showed higher correlation with other subscales than with its own physical functioning. ▪ The Spearman interscale coefficients generally were correlated with each other. Results of known-group comparisons did not show significant differences in terms of disease stage. Regarding education level, patients with high school/university education had better functional scale scores only in certain subscales compared with other subgroups; furthermore, patients with secondary school education had better GH/QoL compared with other subgroups of patients.
Simons [18]	▪ NR	▪ Nausea and vomiting were positively correlated with the reported incidence of nausea as an adverse event (0.126 [*P* = 0.03]) and vomiting (*P* < 0.01); false-positives were negatively correlated (− 0.329 [*P* < 0.01] and − 0.352 [*P* < 0.01], respectively). ▪ Constipation also satisfied criteria for content validity for correspondence (0.189 [*P* < 0.01]) and for lack of false-positives (− 0.394 [*P* < 0.01]). ▪ Diarrhea had a correspondence of 0.292 (*P* < 0.01) and specificity of − 0.260 (*P* < 0.01). ▪ Dyspnea had a correspondence of 0.226 (*P* < 0.01) and a specificity of − 0.349 (*P* < 0.01). ▪ Insomnia failed the criteria. ▪ Upset by hair loss was weakly correlated with alopecia but very specific (− 0.479; *P* < 0.01).
Snyder et al. [19]	▪ The same six QLQ-C30 domains with area under the curve (AUC) values ≥0.70 in the original analysis had AUC values ≥0.70 in the replication sample	▪ Cutoff scores were identified with sensitivity ≥0.84 and specificity ≥0.54
Tan et al. [20]	▪ Cronbach alpha coefficient results for EORTC QLQ-C30 and QLQ-BR23 were 0.846 and 0.873, respectively	▪ The correlation between EORTC QLQ-C30 and EQ-5D QoL instruments demonstrated a modest linear relationship (r = 0.597; *P* < 0.001) that indicated a moderately strong correlation between the two measures
Wallwiener et al. [21]	▪ No differences in terms of acceptance between paper and electronic patient-reported outcome ▪ No significant different in response behavior between paper and electronic patient-reported outcome	▪ NR
Wallwiener et al. [22]	▪ High correlations were shown for both dimensions of reliability (parallel forms reliability and internal consistency) in the patient’s response behavior between paper- and electronic-based questionnaires ▪ Regarding the test of parallel forms reliability, no significant differences were found in 27 of 30 single items and in 14 of 15 scales, whereas a statistically significant correlation in the test of consistency was found in all 30 single items and all 15 scales	▪ NR
Zhang et al. [23]	▪ Cronbach alpha coefficients were close to or greater than 0.7, except for breast symptoms (0.615)	▪ Multitrait scaling analysis demonstrated a good convergent and divergent validity of EORTC QLQ-BR23 and EORTC QLQ-C30 ▪ Using SF-36 as a reference standard to evaluate the dimensions of EORTC QLQ-BR23, most items in EORTC QLQ-BR23 possessed a favorable correlation with its own dimension (r > 0.4) ▪ A statistically significant difference was discovered in dimension scores between patients grouped by ECOG scores except for individual dimensions
**FACT-B**		
Algamdi and Hanneman [24]	▪ Cronbach alpha was 0.91 for the FACT-BA, and 0.43–0.89 for the FACT-BA subscales	▪ NR
Cheung et al. [25]	▪ NR	▪ In a cross-sectional setting, the differences in the effect size favored EQ-5D-5L and the 90% CIs totally fell within the zone that indicated the noninferiority of the EQ-5D-5L (e.g., oncologist-assessed performance status: − 0.26 to 0.04; patient-assessed performance status: − 0.48 to − 0.16; current evidence of disease: − 0.28 to 0.08). In a longitudinal setting, the FACT-B showed larger effect sizes and ICCs than the EQ-5D-5L. The 90% CIs, however, overlapped the noninferiority margin, thus noninferiority in these two aspects could not be confirmed
Jarkovsky et al. [26]	▪ Similar to other validations of FACT-B translations; good reliability, sensitivity, and reliable internal structure after translation	▪ NR
Kobeissi et al. [27]	▪ NR	▪ The following questions were perceived to be most important: ability to meet the needs of my family, pain, emotional support, worry that my condition will get worse, sleep, worry that other family members will get the disease, change in weight, and pain in different areas of the body. ▪ Instrument was perceived to be adequate, appropriate for use, culturally sensitive, simple, and exhaustive.
Lee et al. [28]	▪ For test-retest reliability, the confidence intervals of the differences in ICC overlapped the noninferiority margin	▪ Using performance status, evidence of disease, and treatment status as criteria, the differences (FACT-B minus EQ-5D-3L) in the effect size for discriminative ability were negative or close to, 0 and the 90% confidence intervals (CIs) fell within the zone that indicated noninferiority of EQ-5D-5L ▪ For responsiveness, the CIs of the differences in effect size overlapped the noninferiority margin (difference in effect size (90% CI), FACT-B vs. EQ-5D-5L [0.04 (− 0.79, 0.95])
Matthies et al. [29]	▪ High correlations were shown for both dimensions of reliability (parallel forms reliability and internal consistency) in the patients’ response behavior between paper-based and electronically based questionnaires; regarding the reliability test of parallel forms, no significant differences were found in 35 of 37 single items, while significant correlations in the test for consistency were found in all 37 single items, in all 5 sum individual item subscale scores, and in total FACT-B score	▪ NR
Ng et al. [30]	▪ Cronbach alpha for the FACT-B total score and Trial Outcome Index were 0.91 and 0.87 for the English-speaking sample, respectively; for the Chinese-speaking sample, they were both 0.88 ▪ The ICCs for the FACT-B total score and Trial Outcome Index were 0.82 (95% CI, 0.74–0.87) and 0.84 (95% CI, 0.77–0.89), respectively, for the English-speaking sample; they were 0.88 (95% CI, 0.80–0.93) and 0.89 (95% CI, 0.81–0.94), respectively, for the Chinese-speaking sample	▪ The FACT-B total score and Trial Outcome Index demonstrated known-group validity in differentiating patients with different clinical status. ▪ The English version was responsive to the change in performance status. The Chinese version was shown to be responsive to decline in performance status, but the sample size of Chinese-speaking patients who improved in performance status was too small (*N* = 6) for conclusive analysis about responsiveness to improvement. ▪ Two items concerning sexuality had a high item nonresponse rate (50.2 and 14.4%). ▪ No practically significant difference was found in the total score and in the Trial Outcome Index between the two language versions despite minor differences in 2 of the 37 items. ▪ The English and Chinese versions of the FACT-B are valid, responsive, and reliable instruments in assessing health-related quality of life in Singaporean patients with breast cancer.
Patoo et al. [31]	▪ Internal consistency using Cronbach alpha was 0.63 to 0.93 for the subscales and 0.92 for the total scale	▪ Significant correlations between FACT-B and other measures indicate that this scale had concurrent and discriminant validity. The values of fit indices were satisfactory
**FBSI**		
Lee et al. [32]	▪ For both language versions, the FBSI demonstrated sufficient test-retest reliability (ICC = 0.75–0.77)	▪ For both language versions, the FBSI demonstrated known-group validity and convergent and divergent validity ▪ The English version was responsive to changes in performance status ▪ The Chinese version was responsive to decline in performance status, but there was no conclusive evidence about its responsiveness to improvement in performance status ▪ No practical significant difference was found in the outcomes between the two language versions despite minor difference in one item ▪ The FBSI performed comparably with the FACT-B
**NFBSI-16**		
Garcia et al. [33]	▪ Results provide preliminary support for internal consistency reliability (0.87) of the NFBSI-16	▪ Selected breast cancer–related symptoms and concerns endorsed as high priority by both oncology patients and clinicians for inclusion in the new NFBSI-16, which includes all 8 items from the original FBSI and 8 additional items from FACT-B measures ▪ The NFBSI-16 is formatted by subscale: Disease-Related Symptom, Treatment Side Effect, and General Function and Well-Being ▪ Validity was evidenced by moderate-to-strong relationships with expected criteria
Krohe et al. [34]	▪ NR	▪ All patients for whom data were available demonstrated understanding of the instructions and the recall period of the NFBSI-16 (n = 14/14, 100.0%) and the PROMIS (*n* = 14/14, 100.0%). ▪ > 90% of patients demonstrated understanding of each of the items in the NFBSI-16 and the PROMIS. ▪ > 70% of patients demonstrated understanding of the response options of the NFBSI-16, > 90% understood response options of PROMIS items 1–6, and ≥ 50% understood response options of PROMIS items 7–10. ▪ Conceptual relevance was supported for most items in both questionnaires based on patients’ reports of experiencing the concepts as part of their breast cancer experience.
**YW-BCI36**		
Christophe et al. [35]	▪ Internal consistency (Cronbach alpha values ranging from 0.76 to 0.91) ▪ Temporal reliability (Bravais-Pearson correlations ranging from 0.66 to 0.85)	▪ As expected, there were quite strong correlations between the Young Women With Breast Cancer Inventory and the QLQ-C30 and QLQ-BR23 scores (r ranging from 0.20 to − 0.66), indicating adequate concurrent validity
**Breast Cancer Symptom Scale**		
Horigan et al. [36]	▪ NR	▪ The 9 highest ranked items include: good QoL, maintaining independence, able to sleep, able to concentrate, perform normal activities, being fatigued, having depression, being anxious, and having pain. ▪ The 5 lowest ranked items include: appetite, breast-specific issues, hot flashes, and sexuality. ▪ Ratings by breast cancer subset (newly diagnosed, on treatment, no evidence of disease, hormonal or nonhormonal treatment, metastatic disease, survivors) showed some differences compared with the whole group.
**QLICP-BR**		
Wan et al. [37]	▪ Test-retest reliability for the overall scale and 5 domains are all > 0.75 (overall scale: 0.88) ▪ Internal consistency alpha for each domain is > 0.65, except social domain (0.58)	▪ Most correlation coefficients between each item and its domain are > 0.60. ▪ Overall the correlations between the same and similar domains (between QLICP-BR and QLQ-C30 and QLQ-BR23) are higher than those between different and nonsimilar domains. ▪ The score differences between pretreatment and posttreatment for overall scale, general module, physical domain, psychological domain, and social domain have statistical significance.
**QuEST-Br**		
Harley et al. [38]	▪ Internal consistency was high for all subscales (Cronbach alpha: range, 0.81–0.93) ­ Strenuous activities: 0.87 ­ Everyday tasks: 0.83 ­ Pain: 0.89 ­ Fatigue: 0.93 ­ Impact on activities: 0.88 ­ MHI-5: 0.81 ­ EORTC emotion function: 0.88 ­ Body image: 0.92	▪ Item-convergent validity (item to own scale; correlation corrected for overlap) ­ Strenuous activities: 0.66–0.76 ­ Everyday tasks: 0.55–0.72 ­ Pain: 0.80 ­ Fatigue: 0.81–0.86 ­ Impact on activities: 0.67–0.80 ­ MHI-5: 0.43–0.67 ­ EORTC emotion function: 0.69–0.78 ­ Body image: 0.81–0.86
**INA-BCHRQoL**		
Saptaningsih et al. [39]	▪ Cronbach alpha for physical, cognitive, social, and spiritual domain were higher than 0.8, and the corrected item-total correlation was also higher than 0.3	▪ Each domain of the questionnaire was not influenced by the treatment options. ▪ 24 patients with early stage breast cancer (10 FAC based chemotherapy and 14 taxan-based chemotherapy) were enrolled in the main study, and the score of HRQoL obtained from INA-BCHRQoL was considerably high.
**Unnamed**		
Deshpande et al. [7]	▪ Cronbach alpha value for the questionnaire was 0.93	▪ Patients understood the questionnaire and found the items to be relevant, indicating content validity. ▪ The statistical assessment of the scores did not show the association between scores with age or stage of breast cancer, as sample size was small.
Vanlemmens et al., [9]	▪ Participants reported on 8 dimensions of their quality of life during treatment and follow-up: psychological, physical, family, social, couple, sexuality, domestic, professional, economic	▪ Very few differences were found between the 4 groups (chemotherapy, Herceptin, hormonotherapy, or follow-up) except that patients receiving chemotherapy and patients receiving Herceptin referred more to physical dimension than the group under follow-up
Vanlemmens et al., [8]	▪ Internal consistency (Cronbach alpha) ranged from 0.76 to 0.91 ▪ Test-retest ICC ranged from 0.662 to 0.855	▪ As expected, convergent validity showed strong correlations with quality of life measures (EORTC QLQ-C30).

*ECOG* Eastern Cooperative Oncology Group, *EORTC* European Organization for Research and Treatment of Cancer, *EQ-5D-3L* EuroQoL 3-level 5-dimension, *EQ-5D-5L* EuroQoL 5-level 5-dimension, *FAC* fluorouracil, doxorubicin, and cyclophosphamide, *FACT-B* Functional Assessment of Cancer Therapy-Breast, *FBSI* Functional Assessment of Cancer Therapy-Breast Symptom Index, *HRQoL* health-related quality of life, *ICC* intraclass correlation coefficient, *INA-BCHRQoL* Indonesian Breast Cancer Health-Related Quality of Life, *MHI-5* Mental Health Inventory-5, *NFBSI-16* National Comprehensive Cancer Network-Functional Assessment of Cancer Therapy-Breast Cancer Symptom Index-16, *NR* not reported, *PROMIS* Patient-Reported Outcomes Measurement Information System, *QLICP-BR* Quality of Life Instruments for Cancer Patients-Breast Cancer, *QLQ-BR23* Breast Cancer–Specific Quality of Life Questionnaire-23 item, *QLQ-C30* Quality of Life Questionnaire, Version 3.0, *QoL* quality of life, *QuEST-BR* QuEST Breast Cancer Questionnaire, *SF-36* Short Form Health Survey

### Comparison of item content

A review of the item content of the identified measures reveals variability in the content that each assesses (Table 3). Most of the PROMs assess not only breast cancer symptoms, but the physical and emotional aspects of the disease. Breast cancer–specific symptoms appear not to be included in INA-BCHRQoL, YW-BCI36, and the new measures [7–9]. Physical and emotional or social functioning are included in the QLQ-C30/QLQ-BR23, FACT-B, FBSI, NFBSI-16, QuEST-Br, QLICP-BR, whereas other concepts are addressed within only one or two PROMs. For example, sexual function is included in the QLQ-BR23, but no other PROM; body image is only included in the QLQ-BR23 and the YW-BCI36. Vanlemmens and colleagues’ measure for young women (< 45 years of age) is focused not only on the patient with breast cancer but also on her partner and their relationship (i.e., couple cohesion, managing children/everyday life) [8]. Regarding the financial impact of living with breast cancer, only the measure by Vanlemmens et al. [8] and the YW-BC136 include this concept. Approximately two thirds of the identified articles (22/33) focused on the EORTC QLQ-C30, EORTC QLQ-BR23, or the FACT-B.

### EORTC QLQ-C30 and EORTC QLQ-BR23

Fourteen publications provided additional psychometric validation of the EORTC measures (QLQ-C30 and QLQ-BR23) (Table 4). Of these, eight were focused on validation of the EORTC modules in new languages (e.g., Chinese [23], Arabic [10, 11, 14, 20], Mexican-Spanish [13] or confirmation that the measures was understood by or used in larger populations (Brazil [16], Albania [17], Singapore [20]); two articles presented additional confirmation of content validity [18, 19], one article tested the replicability of cutoff scores [19], and three demonstrated acceptability of the measures in ePRO platforms [15, 21, 22].

Internal consistency was assessed using the Cronbach alpha coefficient for translations. The breast symptoms scale for several translations (Chinese [23], Arabic [14], and Mexican-Spanish [13]) was below 0.70; otherwise reliability of the QLQ-BR23 translations met established criteria (Table 5). Test-retest reliability was also established for the Arabic version [10]. Various methods were used to evaluate the validity of the translations, including multitrait scaling and known-groups comparisons. Item-convergent validity was demonstrated (i.e., exceeding the 0.40 criterion) [14, 17, 23]. The questionnaires differentiated patients with lymphedema from those without [29], differentiated patients with early stage breast cancer versus those with locally advanced breast cancer [11, 13], and were responsive to changes following treatment [13]. Additional content validity for signs and symptoms was evaluated by testing the correlation between reported adverse events and responses to the QLQ-C30 [18].

Bjelic-Radisic et al. [12] evaluated whether updates in breast cancer treatment necessitate updating the EORTC QLQ-BR23, which was developed in 1996. A literature review and interviews with patients and health care providers suggest that additional concepts were missing. The new items contain two multi-item scales: target symptom scale (20 items) and satisfaction scale (2 items). The target symptom scale can be further divided into three subscales: endocrine therapy scale, endocrine sexual scale, and skin/mucosa scale. Further psychometric validation is underway.

Administration of the measures via ePRO also has demonstrated reliability and validity [15, 21].

### FACT-B

The majority of the FACT-B publications presented reliability and validity data for translations of the measure into Arabic [24], Persian [31], Czech [26], Lebanese Arabic [27], and Chinese [30] (Table 4). One publication presented data regarding the appropriateness of an ePRO application [29]. Two articles compared the properties of the FACT-B (disease-specific measure) with that of a general HRQoL measure, the EQ-5D [25, 28].

Internal consistency reliability (Cronbach alpha) and test-rest reliability (intraclass correlation coefficient) met accepted thresholds for the translations [24, 26, 31] (Table 5). Content validity was established in Lebanese Arabic translation [27]. Administration of the measure via ePRO was deemed acceptable [29].

### Other measures

#### FBSI and NFBSI-16

The Chinese translation of the FBSI has demonstrated adequate test-retest reliability as well as known-group validity and convergent and divergent validity [32]. Garcia et al. [33] sought to develop a new version of the FBSI in accordance with US Food and Drug Administration guidance for PRO measures that provides assessment on a symptom level and improves upon the original FBSI by emphasizing input from patients. Specifically, 52 patients with breast cancer provided their top-priority symptoms/concerns through open-ended interviews and symptom checklists. After patient input was reviewed, eight additional items were added to the original FBSI, creating the NFBSI-16. Conceptual relevance was supported for most items in the NFBSI-16 based on patients’ reports of experiencing the concepts as part of their breast cancer experience [34].

#### YW-BCI36

Christophe et al. [35] developed a questionnaire specifically measuring the subjective experience of nonmetastatic breast cancer in young women (aged 45 years or younger when diagnosed), their perceptions regarding its treatment in their daily life, and the repercussions of the disease. Reliability and validity of the new measure were demonstrated (Table 5).

#### BCSS

Horigan et al. [36] conducted a large survey of registered patients with breast cancer to further document the content validity of the BCSS. Specifically, the patients were asked to rank 21 issues identified as important to them. The nine highest ranked items include good QoL, maintaining independence, able to sleep, able to concentrate, perform normal activities, being fatigued, having depression, being anxious, and having pain. The five lowest ranked items include appetite, breast-specific issues, hot flashes, and sexuality. Ratings by breast cancer subset (newly diagnosed, on treatment, no evidence of disease, hormonal or nonhormonal treatment, metastatic disease, survivors) showed some differences compared with those by the whole group.

#### QuEST-Br

Harley et al. [38] adapted existing HRQoL instruments (EORTC measures) for use in routine clinical practice delivering outpatient chemotherapy for breast cancer. Methods followed the guidelines laid out by the EORTC Quality-of-Life Group for developing questionnaire modules [40]. Internal consistency reliability was > 0.70 for the QuEST-Br scale [38].

#### QLICP-BR

Wan et al. [37] developed and validated a QoL instrument for patients with breast cancer in China. The measure was developed with particular attention to Chinese culture. For example, the family relationship and kinship play very important roles in daily life. Taoism and traditional medicine focus on good temper and high spirit. Good appetite, sleep, and energy are highly regarded in daily life, and food culture is very important [37]. The QLICP-BR was found to have adequate reliability and validity (Table 5).

#### INA-BCHRQoL

Saptaningsih et al. [39] developed a new measure to capture not only the physical, cognitive, and psychological aspects of patients but also the spiritual aspect. The questionnaire was developed in Indonesia and was designed to be culturally relevant (i.e., it included a spiritual domain, which is suitable for Indonesia, as it is a very religious country) to the breast cancer population in Indonesia. The INA-BCHRQoL was found to have adequate reliability and validity (Table 5).

#### Unnamed measures

Deshpande et al. [7] developed and validated a patient-reported questionnaire to assess the QoL outcomes of patients with breast cancer in India. Reliability and content validity were demonstrated (Table 5).

Vanlemmens et al. [8, 9] developed and validated a particular and specific inventory for measuring the impact of breast cancer on the QoL of young women (< 45 years of age) with nonmetastatic disease and the QoL of their partners. Reliability (internal consistency and test-retest) has been established. Convergent validity showed strong correlations with QoL measures (EORTC QLQ-C30) (Table 5).

## Discussion

Understanding the effect of breast cancer treatment on a patient’s HRQoL is a central clinical and research question. However, to accurately assess HRQoL, valid and reliable PROMs are needed: that is, PROMs with evidence of reliability, validity, and responsiveness in the population of interest (breast cancer). This review sought to identify disease-specific HRQoL measures with evidence of validation in the breast cancer population for potential use in patients underwent systemic treatment for breast cancer (excluding surgery and radiotherapy). In addition to the EORTC QLQ-C30, QLQ- BR23, and FACT-B, we identified an additional nine potential measures.

The identified PROMs vary in the content that they assess. For example, Vanlemmens and colleagues’ measure for young women (< 45 years of age) is focused not only on the patient with breast cancer but also on her partner and their relationship (i.e., couple cohesion, managing children/everyday life) [9]. This measure also assesses impact of breast cancer on the woman’s career and finances. Other than the YW-BCI36, none of the other instruments assess the impact of breast cancer on a woman’s career or finances. Conversely, most do assess not only breast symptoms but also the physical and emotional/psychological impact of disease.

The YW-BCI36 [35] and the measure developed by Vanlemmens et al. [8, 9] were both developed specifically for women < 45 years old with breast cancer. Younger women with breast cancer have concerns (i.e., childcare, financial) that older women with breast cancer may not, thus these measures were developed specifically for this population. Several other PROMs were developed to meet a specific unmet need within regions (China, Indonesia, India) for measures that were culturally appropriate (QLICP-BC [37], INA-BCHRQoL [39], Indian breast cancer measure [7]). Given that these PROMs have been developed to be culturally relevant for a specific region/population, they may not be appropriate for global studies.

Psychometric qualities that may be examined in the evaluation of an instrument may include acceptability, validity, reliability (including internal consistency and test-retest reliability), and responsiveness. When questionnaire responsiveness (the ability of a scale to detect significant change over time, assessed by comparing scores before and after an intervention of known efficacy) was examined on the basis of various methods, including *t* tests, effect sizes, standardized response means, or responsiveness statistics, the information available was scarce.

The FACT-B and the QLQ-BR23 were designed for use in patients with breast cancer with a range of disease stages and undergoing different treatments. The EORTC QLQ-BR23 and the FACT-B are well developed instruments that have been extensively tested among patients with breast cancer. The FACT-B is shorter than the QLQ-BR23 and covers fewer symptoms and treatment-related side effects. This review has identified additional translations of the measures, providing further evidence of their validity. Internal consistency estimates of reliability were adequate for research purposes, although the internal consistency estimates were somewhat lower for the cognitive and breast symptoms scales. Further psychometric testing of the Breast Cancer–Specific Quality of Life Questionnaire-45 item (QLQ-BR45) may provide improved results. A recent publication [41] provides more detailed updates on the development of the QLQ-BR45. The QLQ-BR23 was one of the first disease-specific questionnaires developed in 1996 to assess QoL in patients with breast cancer. Given the effects of newer therapeutic options available since then, the developers believed it was evident that the original 23-item QLQ-BR23 may not be able to cover many important QoL issues and potential side effects of newer treatments. Therefore, the EORTC Quality of Life Group decided to update this module, eventually creating the QLQ-BR45. The development of the QLQ-BR45 involved a systematic literature review to identify relevant QOL issues for patients, interviews with patients and providers, and pretesting of a preliminary module in an international phase 3 study. Results of the literature review and discussions with patients and providers indicated that the original QLQ-BR23 inadequately covered concepts currently relevant to patients with breast cancer. Thus, new items were developed (added to the existing QLQ-BR23) and pretested in a multinational study, resulting in the QLQ-BR45, which is currently undergoing further psychometric testing.

Much of the additional psychometric data for the EORTC QLQ-C30, QLQ-BR23, and FACT-B are from new translations, further confirming the acceptability of these measures. Reliability of tablet or ePRO versions of the measures were also confirmed. New instruments were developed de novo in order to be considered more culturally relevant to patients in Asian countries. While these measures have demonstrated adequate internal consistency, test-retest and responsiveness data are lacking.

## Conclusions

Even though, historically there have been limited options for validated measures to assess HRQoL in breast cancer patients, a number of new options for assessing HRQoL in breast cancer population have been developed and validated in recent years. This review supports the reliability and validity of the EORTC QLQ-C30 and FACT-B; new translations and electronic versions of these measures further support their use for this population. Researchers should ensure that their selected PROMs are suitable for their target patient population, anticipated line of therapy, and the expected side effects of the therapies involved.

## Data Availability

All data generated or analyzed during this study are included in this published article [and its supplementary information files].

## Abbreviations

BCSS: Breast Cancer Symptom Scale
EORTC: European Organization for Research and Treatment of Cancer
ePRO: Electronic patient-reported outcome
FACT-B: Functional Assessment of Cancer Therapy-Breast
FBSI: Functional Assessment of Cancer Therapy-Breast Symptom Index
HRQoL: Health-related quality of life
INA-BCHRQoL: Indonesian Breast Cancer Health-Related Quality of Life
NFBSI-16: National Comprehensive Cancer Network-Functional Assessment of Cancer Therapy-Breast Cancer Symptom Index-16
PICOS: Population, intervention, comparison, outcome, study type
PRO: Patient-reported outcome
PROM: Patient-reported outcome measure
QLICP-BR: Quality of Life Instruments for Cancer Patients-Breast Cancer
QLQ-BR23: Breast Cancer–Specific Quality of Life Questionnaire-23 item
QLQ-BR45: Breast Cancer–Specific Quality of Life Questionnaire-45 item
QLQ-C30: Quality of Life Questionnaire, Version 3.0
QoL: Quality of life
QuEST-Br: QuEST Breast Cancer Questionnaire
YW-BCI36: Young Women with Breast Cancer Inventory
